# Supplementary material for: Neuroplastin Expression in Male Mice Is Essential for Fertility, Mating, and Adult Testosterone Levels
Source: Int J Mol Sci. 2023 Dec 22;25(1):177. doi: 10.3390/ijms25010177 (PMC10779036; doi:10.3390/ijms25010177)
Supplement: Supplementary file 1 [file ijms-25-00177-s001.zip › ijms-2725420-supplementary.pdf]

Testis weight [g]

age [days]	Nptn+/+	Nptn+/+	Nptn+/+	Nptn+/+	Nptn+/+	Nptn+/+	Nptn-/-	Nptn-/-	Nptn-/-	Nptn-/-	Nptn-/-	Nptn-/-
20	20	9	21	8	44	5	20	13	20	36		
28	42	28	44	54	16	63	31	39	41	41		
34	57	56	37	63	53	39	59	64	35	36	26	19
45	78	89	69	96	78	101	28	67	101	111	34	98
90	191	205	188	148	189		163	165	187	170	189	

cells percent [%]

	Nptn+/+ individuals						Nptn-/- individuals					
**age [days]**	1c Nptn+/+	1c Nptn+/+	1c Nptn+/+	1c Nptn+/+	1c Nptn+/+	1c Nptn+/+	1c Nptn-/-	1c Nptn-/-	1c Nptn-/-	1c Nptn-/-	1c Nptn-/-	1c Nptn-/-
**20**	8,8	10,2	4,5	4,8	4,9	5,4	14,5	6	8,1	5,9		
**28**	51	32,8	13,9	44,7	45,1	44,4	46,2	55,2	49,2	52,5		
**34**	71,3	69,7	59,9	66,6	57,9	27	67,1	62,1	65,6	64,6	54,1	53,9
**45**	79,8	74,5	75,7	75,5	75,6	48	66,8	72,1	69,4	72	75,6	75,9

	Nptn+/+ individuals						Nptn-/- individuals					
**age [days]**	2c Nptn+/+	2c Nptn+/+	2c Nptn+/+	2c Nptn+/+	2c Nptn+/+	2c Nptn+/+	2c Nptn-/-	2c Nptn-/-	2c Nptn-/-	2c Nptn-/-	2c Nptn-/-	2c Nptn-/-
**20**	37,7	45,1	49,3	49,5	45,2	49,3	31,7	48,8	43	45,3		
**28**	20	27,9	46,5	31,1	25,5	27	23,7	20,5	21,8	20,4		
**34**	11	12,9	17,4	14,4	18	30,3	13,4	15,5	16,3	17,9	26,9	26,2
**45**	10,8	13,3	12,3	12,5	12,3	32	18,2	13	14,3	14,1	12,2	12,3

	Nptn+/+ individuals						Nptn-/- individuals					
**age [days]**	s Nptn+/+	s Nptn+/+	s Nptn+/+	s Nptn+/+	s Nptn+/+	s Nptn+/+	s Nptn-/-	s Nptn-/-	s Nptn-/-	s Nptn-/-	s Nptn-/-	s Nptn-/-
**20**	4,3	4,7	5,6	6,4	5,2	5,9	3,6	5	5,3	5,7		
**28**	3,6	5	6,1	5,1	5,3	3,8	4,2	3,1	3,5	3,1		
**34**	2,3	2,5	3,4	2,6	3,2	4,7	2,9	3,1	4	4,2	4,9	5,4
**45**	2,7	3,4	3,4	3	3,8	5,4	3,3	3	4	3,5	3,5	3,3

	Nptn+/+ individuals						Nptn-/- individuals					
**age [days]**	4c Nptn+/+	4c Nptn+/+	4c Nptn+/+	4c Nptn+/+	4c Nptn+/+	4c Nptn+/+	4c Nptn-/-	4c Nptn-/-	4c Nptn-/-	4c Nptn-/-	4c Nptn-/-	4c Nptn-/-
**20**	48,8	39,5	40,2	38,8	44,1	39	49,7	39,8	43,1	42		
**28**	24,6	33,2	32,9	17,9	21,9	23,3	25,4	20,6	25	23,7		
**34**	14,9	14	18,4	16	20,5	36,9	16	18,5	11,7	10,7	13,3	11,4
**45**	6	7,3	7,4	7,9	6,3	12,8	10,8	6,7	9,2	7,3	8	5,7
